# Transient kinetics of aminoglycoside phosphotransferase(3′)-IIIa reveals a potential drug target in the antibiotic resistance mechanism

**DOI:** 10.1016/j.febslet.2012.10.027

**Published:** 2012-11-30

**Authors:** Perrine Lallemand, Nadia Leban, Simone Kunzelmann, Laurent Chaloin, Engin H. Serpersu, Martin R. Webb, Tom Barman, Corinne Lionne

**Affiliations:** a Centre d’études d’agents Pathogènes et Biotechnologies pour la Santé (CPBS), UMR 5236 CNRS, University Montpellier I & II, 1919 Route de Mende, 34293 Montpellier Cedex 5, France; b Division of Physical Biochemistry, MRC National Institute for Medical Research, The Ridgeway, Mill Hill, London NW7 1AA, United Kingdom; c Department of Biochemistry and Cellular and Molecular Biology, University of Tennessee, Knoxville, TN 37996, USA; d Volunteer, 8 rue Dom Vaissette, 34000 Montpellier, France

**Keywords:** APH(3′)-IIIa, aminoglycoside phosphotransferase(3′)-IIIa (EC 2.7.1.95), KanA, kanamycin A, MDCC-ParM, ParM labeled with *N-*[2-(1-maleimidyl)ethyl]-7-diethylaminocoumarin-3-carboxamide, Quench flow, Stopped flow, Pre-steady state kinetics, Uncompetitive inhibition, Abortive complex, Dead end product

## Abstract

Aminoglycoside phosphotransferases are bacterial enzymes responsible for the inactivation of aminoglycoside antibiotics by *O*-phosphorylation. It is important to understand the mechanism of enzymes in order to find efficient drugs. Using rapid-mixing methods, we studied the transient kinetics of aminoglycoside phosphotransferase(3′)-IIIa. We show that an ADP-enzyme complex is the main steady state intermediate. This intermediate interacts strongly with kanamycin A to form an abortive complex that traps the enzyme in an inactive state. A good strategy to prevent the inactivation of aminoglycosides would be to develop uncompetitive inhibitors that interact with this key ADP-enzyme complex.

## Highlights

► APH(3′)-IIIa is a bacterial kinase responsible for antibiotics resistance. ► Release of ADP is the rate limiting step of Kanamycin A phosphorylation. ► An ADP-enzyme complex is the main steady state intermediate. ► A strategy to prevent the phosphorylation of aminoglycosides is uncompetitive inhibition. ► A good target for uncompetitive inhibitors is the ADP-enzyme complex.

## 1 Introduction

Bacterial resistance to antibiotics is a major concern in medical treatment. Bacteria have evolved several mechanisms to combat antibiotics [1]; these are often enzyme systems that are expressed upon antibiotic challenge. A strategy for combating bacterial antibiotic resistance is to develop drugs that inhibit specifically these enzymes. This is not an easy matter: as pointed out by Duclert-Savatier et al. [2], to evaluate the pharmacological or medical importance of an enzyme, one must have a clear understanding of its function and, especially, its reaction pathway and mechanism of action. As an example, this understanding is important for the rational design of inhibitors of enzymes such as the aminoglycoside transferases that certain bacteria produce to inactivate aminoglycoside antibiotics [3]. Cornish-Bowden [4] proposed that uncompetitive inhibitors, that is to say inhibitors that interact specifically with an intermediate on an enzyme reaction pathway, are especially powerful inhibitors. Thus, he proposes that once the enzyme has started to turn over*,* such inhibitors have a catastrophic effect on its activity.

The aminoglycosides are especially susceptible to bacterial resistance. To protect them against this type of antibiotic, several bacteria have evolved enzymes that lead to their inactivation by chemical modification such as *N*-acetylation, *O*-adenylation and, in particular, *O*-phosphorylation [5–7].

Aminoglycoside phosphotransferase(3′)-IIIa, APH(3′)-IIIa, has a broad antibiotic specificity and has been the subject of several structural and steady state kinetic studies [8–12]. With kanamycin A (KanA) and ATP as substrates, this enzyme catalyzes the reaction:

$$\text{KanA}+\text{ATP}↔3^{\prime }\text{-phosphoKanA}+\text{ADP}$$

In a steady state study in which ADP production was followed by an indirect method using coupled enzyme system, McKay and Wright [10,11] proposed that on the reaction pathway of APH(3′)-IIIa, 3′-phosphoKanA is released rapidly followed by a slow release of ADP with kinetics that limit the overall reaction. Here, we tested this hypothesis by a transient kinetic study on APH(3′)-IIIa. We exploited two methods: rapid quench flow by which reaction mixtures are sampled on the millisecond to second time scale [13] and stopped flow which allows reactions to be followed continuously by optical methods [14]. We show unambiguously that ADP release is the rate limiting step of the reaction pathway and that KanA is at once a substrate and a powerful inhibitor because it interacts with the intermediate E·ADP as well as with the apoenzyme.

## 2 Materials and methods

### 2.1 Materials

KanA sulphate, ADP, ATP (BioXtra), phospho(enol)pyruvic acid and pyruvate kinase/lactic dehydrogenase enzymes from rabbit muscle were from Sigma–Aldrich. NADH was from Roche. Recombinant APH(3′)-IIIa from *Enterococcus faecalis* was produced in *Escherichia coli* BL21 (DE3) transformed with pET15b plasmid encoding for APH(3′)-IIIa with a 6His-tag in N-terminal (from E.H. Serpersu). Production and purification procedures were as in [15] except that the induction was at 20 °C OVN. The fractions containing APH(3′)-IIIa were concentrated to 40–60 mg/ml in 20 mM Tris–HCl pH 7.5, 100 mM NaCl, 1 mM DTT using an ultra-filtration device (Amicon® Ultra-15, cutoff 10 kDa, Millipore). Aliquots of 99% pure protein were stored at −20 °C with 50% glycerol and 10 mM DTT. The concentration of APH(3′)-IIIa was determined using an extinction coefficient of 48 735 M^−1^ cm^−1^ at 280 nm.

Except otherwise stated, in the text APH(3′)-IIIa refers to the 6His-tagged recombinant APH(3′)-IIIa. As a control, the removal of the N-terminal 6His-tag was carried out by cleavage of the tagged protein with thrombin as in [16]. The catalytic activity was assayed and compared to that of fresh 6His-tagged APH(3′)-IIIa and of the protein handle in the same way as the one cleaved (except the presence of thrombin). The 6His-tag and the presence of sulphate at 0.2 mM (KanA sulphate used) had no effect on the steady state rates (not shown).

### 2.2 Experimental conditions

Experiments were carried out at 25 °C and in a buffer containing 50 mM Tris–HCl pH 7.5, 40 mM KCl and 1 mM free MgCl_2_. The concentration of free Mg^2+^ used was 1 mM and not 10 mM as in main studies with APH(3′)-IIIa because 1 mM is rarely inhibitory with kinases and is probably close to the *in vivo* level [17]. Equimolar concentration of MgCl_2_ was added with ADP and ATP. In the text, ADP and ATP refer to MgADP and MgATP, respectively. The concentrations of reactants given refer to the final reaction mixture concentrations.

### 2.3 ADP measurements and transient kinetic methods

The time courses of ADP production were obtained by three methods.

The *quench flow method* [13,18] is essentially a chemical sampling method on the time scale of milliseconds to several seconds. The experiments were carried out in a thermostated beaker or a QFM-400 (Bio-Logic, France) thermostatically controlled equipment. APH(3′)-IIIa, pre-incubated with KanA, was mixed with ATP in the apparatus, the reaction mixtures aged for specific times, quenched in acid (10% perchloric acid) and ADP measured by HPLC as in [19]. By this method, the total concentrations of ADP, that is to say enzyme-bound as well as free ADP, were obtained.

In the *coupled assay method* of McKay and Wright [10], free ADP production was measured as NADH consumption by coupling the APH reaction to the pyruvate kinase/lactate dehydrogenase system. Experiments were carried out in a thermostatically controlled SF-61 DX2 stopped flow apparatus (TgK Scientific, UK). APH(3′)-IIIa pre-incubated with KanA (or ATP), 140 μM NADH, 2 mM phospho(enol)pyruvate, 16 U/ml pyruvate kinase and 23 U/ml lactate dehydrogenase was mixed with ATP (or KanA) in the apparatus and the absorbance at 340 nm was measured as a function of time. The concentration of NADH was determined using an extinction coefficient of 6 220 M^−1^ cm^−1^ at 340 nm.

In the *ADP biosensor method*, free ADP was measured by the use of an engineered bacterial actin homologue labelled with a single coumarin fluorophore, MDCC-ParM [20]. Fluorescence stopped flow experiments were carried out as in [15]. The excitation wavelength was 436 nm and the emission wavelength was >455 nm using a cut-off filter. The excitation and emission slits were 2 nm. APH(3′)-IIIa, pre-incubated with 100 μM KanA, was mixed in the apparatus with ATP and 30 μM MDCC-ParM and the fluorescence was measured as a function of time. The background signal coming from ATP binding to MDCC-ParM was corrected for by measuring the background fluorescence time course by mixing the same solutions but without KanA. The concentration of free ADP was determined by measuring the total fluorescence change (at the end of the reaction), assuming that all ATP has been converted to ADP (see below) that is then trapped by MDCC-ParM [20].

## 3 Results

### 3.1 Steady state experiments

**Fig. 1:**
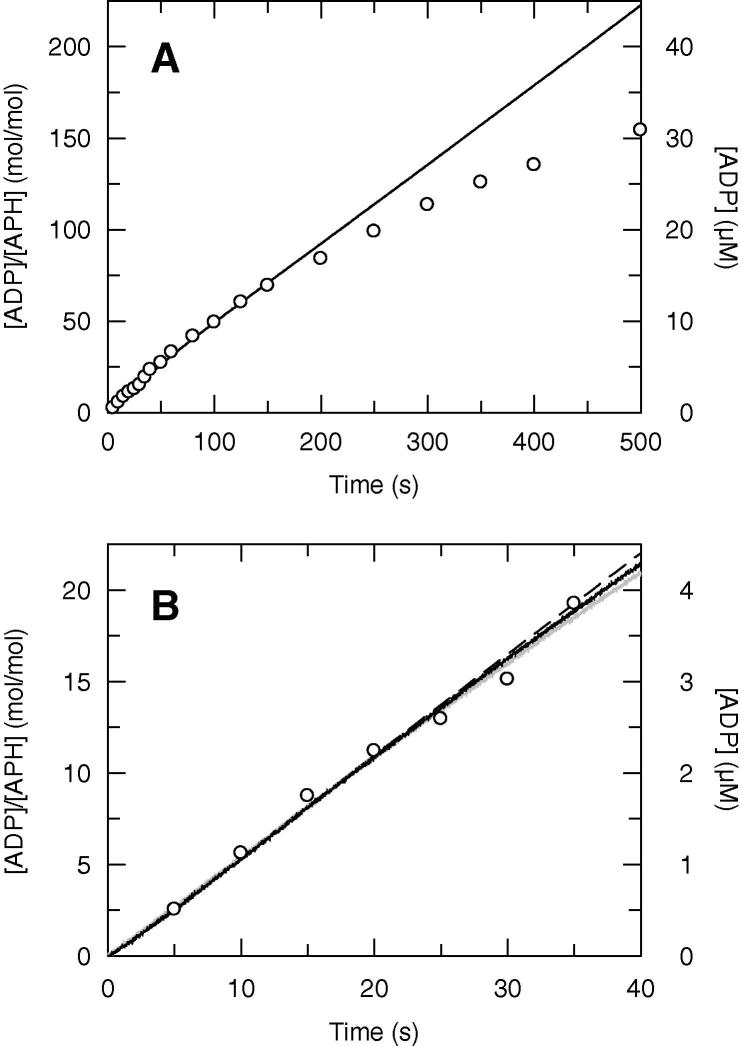
Steady state time courses of ADP production measured by the coupled assay (absorbance stopped flow) and the quench flow methods on two different time scales: 500 s (A) and 40 s (B). The reaction mixtures were 0.2 μM APH, 100 μM KanA and 200 μM ATP. Stopped flow traces are shown as continuous lines and quench flow as circles. In B, stopped flow traces were obtained either by mixing APH and KanA with ATP (black line) or APH and ATP with KanA (grey line). In quench flow, APH was pre-incubated with KanA and mixed with ATP in the apparatus. The fitting of the linear portion of the quench flow time course is shown as a dashed line.

A comparison of the steady state time courses of ADP production measured by the coupled assay and the quench flow methods is shown in Fig. 1. Whereas the time course obtained by the coupled assay method is linear over 500 s (Fig. 1A), that obtained by the quench flow method is not, and only the very initial portion (Fig. 1B) was used to obtain the steady state rate (*k*_ss_). The initial *k*_ss_ determined by the two methods were identical: 0.55 ± 0.02 s^−1^ by quench flow, 0.54 ± 0.01 s^−1^ by stopped flow. The order of mixing of APH with the two substrates had no effect on *k*_ss_: 0.54 ± 0.01 s^−1^ when pre-incubating APH with KanA and then mixing with ATP compared to 0.53 ± 0.01 s^−1^ when pre-incubating APH with ATP and mixing with KanA (black line and grey line respectively in Fig. 1B). This suggests that *k*_ss_ is not limited by the binding of substrates.

The non-linearity of the quench flow time course is almost certainly due to the accumulation of ADP that may compete with ATP for the binding to the active site. In the coupled assay method, the ADP is removed as soon as it is formed and converted back to ATP by pyruvate kinase. We conclude that whereas the chemical sampling method is simpler, more direct and more flexible than the linked enzyme assay method, which does not allow for transient kinetic studies, care must be taken to consider only the initial time course.

**Fig. 2:**
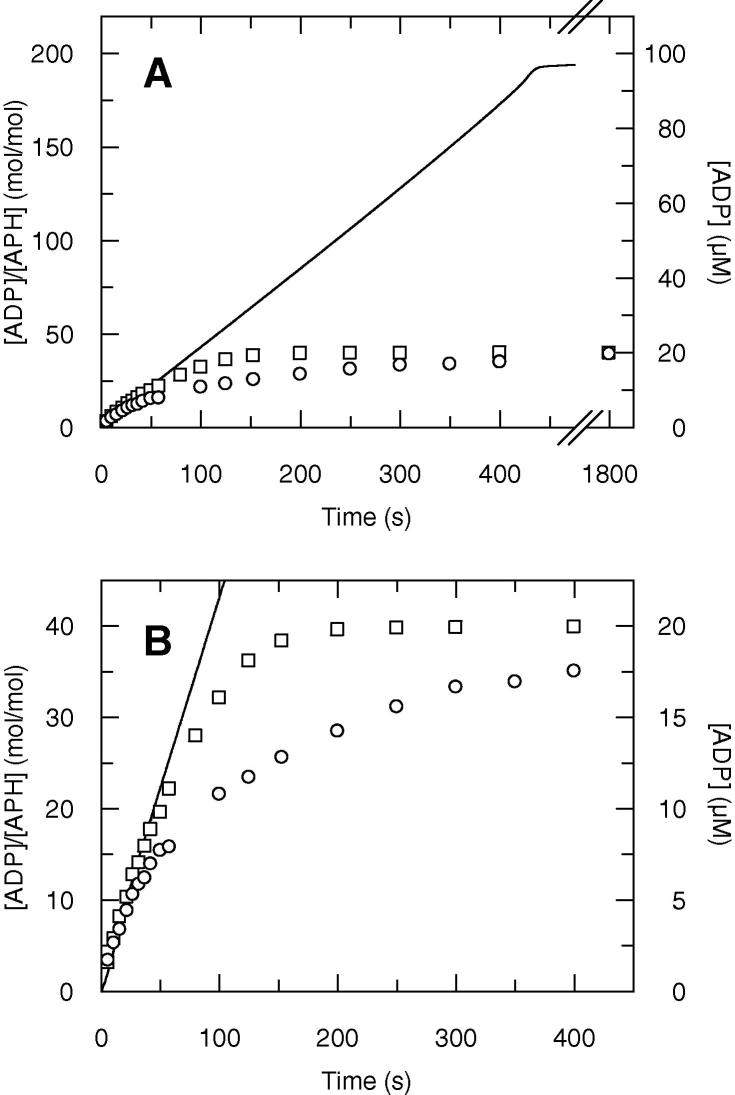
Steady state time courses of ADP production measured by the coupled assay (absorbance stopped flow, continuous line) and the quench flow methods in the absence (circle) or presence (squares) of 30 μM MDCC-ParM. Time courses are shown on two different time scales: 30 min (A) or 400 s (B). The reaction mixtures were 0.5 μM APH, 100 μM KanA and 20 μM ATP.

To confirm this, we carried out steady state measurements by the quench flow method (which measures total ADP) in the presence or not of 30 μM MDCC-ParM, an ADP biosensor [20] that traps the free ADP as soon as it is released. At this concentration of MDCC-ParM, binding of ADP is fast and tight, as the *K*_d_ is <1 μM [20]. Because MDCC-ParM has to be in excess compared to the ADP produced by the APH catalysed reaction, this experiment was done at relatively low ATP concentration (20 μM). As a comparison, the experiment was carried out in the same conditions in a stopped flow apparatus, using the coupled assay method. The time courses obtained are shown in Fig. 2. Again, the time course obtained by the coupled assay method is linear, but that obtained by the quench flow method is not. This non-linearity may result either from the inhibition of the reaction by the ADP released or by the decrease of ATP concentration below the *K*_m_ (10 μM, data not shown). Removing the free ADP by MDCC-ParM significantly increases the duration of the initial linear portion of the time course. The later curvature may be explained by the progressive depletion of ATP.

With the three methods, the reaction appears to go to completion (Fig. 2A) which shows that the overall forward reaction of APH(3′)-IIIa is virtually irreversible. In the quench flow experiments, the final plateau corresponds to all ATP being converted into ADP (20 μM). In the coupled system experiment, the ATP is constantly regenerated from ADP and therefore the reaction stops when all KanA has been used (100 μM).

### 3.2 Transient kinetics of ADP formation

Both APH(3′)-Ia and APH(3′)-IIa have significant ATPase activities [21]. Whereas in 1996, McKay and Wright [11] found that APH(3′)-IIIa had no ATPase activity, earlier in the same group [22] and in our hands it had. In our conditions and at 20 μM ATP, the ATPase steady state rate was 0.0017 s^−1^ which is 0.66% of the kinase activity at the same concentration of ATP (not shown). Whereas this low activity had little effect on the steady state kinetics, it precluded transient kinetic experiments (that is to say at high enzyme concentrations) in which APH(3′)-IIIa is mixed with ATP before KanA.

**Fig. 3:**
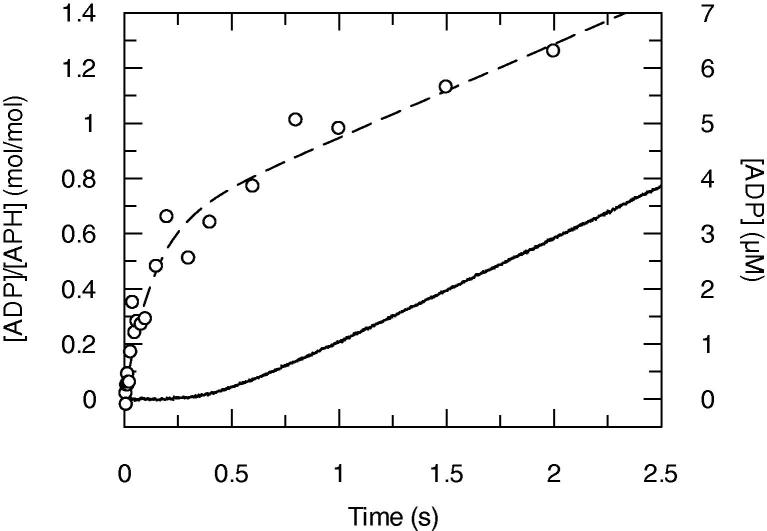
Transient time courses of free and total ADP production measured by the ADP biosensor (fluorescence stopped flow) and the quench flow methods. The reaction mixtures were 5 μM APH, 100 μM KanA and 20 μM ATP, plus 30 μM MDCC-ParM in the fluorescence stopped flow experiment. The stopped flow trace is shown as a continuous line and the quench flow as circles with a single exponential plus linear fit as a dashed line.

Two typical time courses of ADP formation with resolution in the milliseconds range are illustrated in Fig. 3. The *total ADP* time course was obtained by the rapid quench flow method and it shows a transient burst of ADP formation, followed by a linear rise in ADP. This burst suggests that ADP, free or enzyme-bound, accumulates before the steady state is reached. The time course fits to an exponential phase of rate constant *k*_burst_ = 7.1 ± 1.4 s^−1^ and amplitude *A*_burst_ = 0.64 ± 0.06 mol ADP/mol enzyme, that is followed by the steady state of rate constant *k*_ss_ = 0.31 ± 0.04 s^−1^. When the enzyme on its own was mixed with KanA and ATP together, the time course of total ADP was identical to that in Fig. 3 (time course not illustrated).

To determine whether or not the burst is due to enzyme-bound or free ADP, we studied the reaction by using the ADP biosensor [20] to measure the kinetics of ADP release. The *free ADP* time course was obtained by fluorescence stopped flow (Fig. 3). This shows a lag followed by a linear rise in free ADP. The linear part (from 1 s) fits well to a straight line that represents *k*_ss_ = 0.37 ± 0.01 s^−1^, which is in good agreement with the 0.31 s^−1^ estimated by the quench flow method. The intercept of the linear fit with the *X* axis gives an estimate for the duration of the lag phase of 430 ms. The absence of a burst of free ADP shows that the total ADP burst obtained by the rapid quench flow method is due to enzyme-bound ADP. It other words, ADP release is the rate limiting step of the reaction pathway. The transient lag phase preceding the steady state in the fluorescence time course is presumably the manifestation of the time required for the formation of enzyme-bound ADP.

### 3.3 Effect of added ADP on the APH(3′)-IIIa reaction

**Fig. 4:**
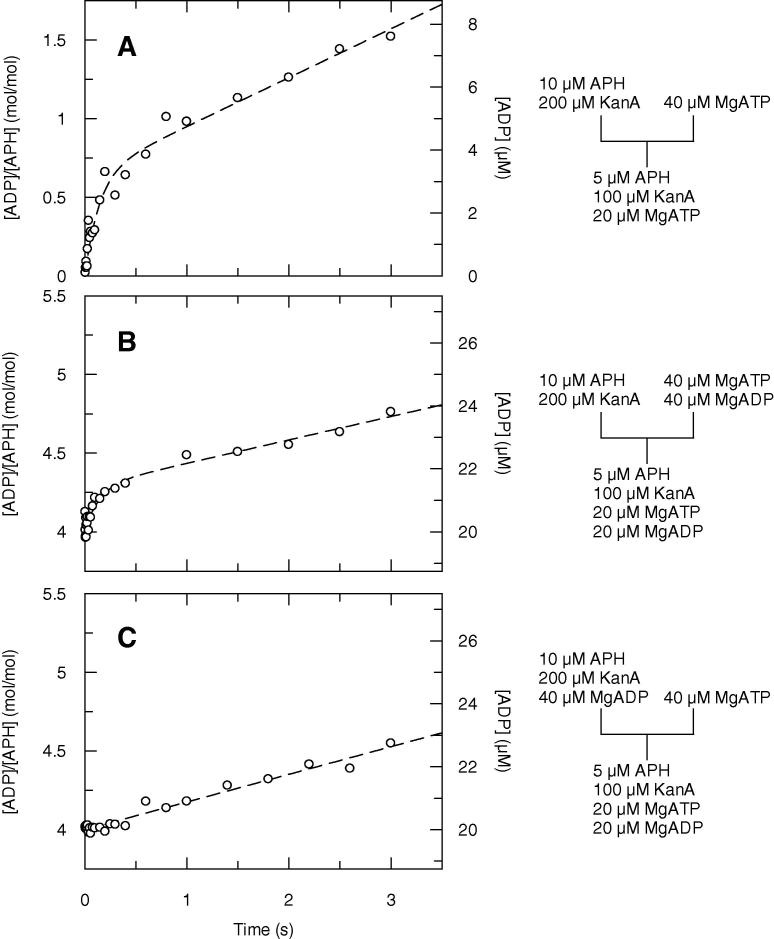
Transient time courses of total ADP production measured by the quench flow method. The reaction mixtures were 5 μM APH, 100 μM KanA and 20 μM ATP (A) plus 20 μM ADP that had been pre-incubated with ATP (B) or APH and KanA (C).

As illustrated in Fig. 4, three quench-flow experiments were carried out. Fig. 4A represents the control experiment, that is to say a time course in the absence of added ADP (also see Fig. 3). In Fig. 4B, the ATP had been incubated with equimolar concentration of ADP before mixing with APH and KanA. This gives a time course with *A*_burst_ = 0.29 ± 0.05 mol/mol, *k*_burst_ = 7.1 ± 2.9 s^−1^ and *k*_ss_ = 0.15 ± 0.03 s^−1^. We explain the ∼50% reduction of both *A*_burst_ and *k*_ss_ by the competition between ADP and ATP for the binding to E·KanA. The enzyme in the abortive E·KanA·ADP complex cannot turn over on the time scale of the transient burst because the required release of ADP is slow (see below). The remaining portion of enzyme appears to be fully active because the *k*_burst_ obtained is close to that in the absence of added ADP.

In Fig. 4C, the E·KanA complex had been pre-incubated with ADP before mixing with ATP. We underline that the concentrations in the final reaction mixtures were identical to those in Fig. 4B. Thus, the steady state rates were similar (0.15 ± 0.03 s^−1^ in Fig. 4B and 0.17 ± 0.02 s^−1^ in Fig. 4C). However, the mixing of E·KanA with ADP before ATP has a catastrophic effect on the pre-steady state kinetics: there was no sign of a transient burst phase of ADP. This is evidence that at this concentration of ADP in the pre-incubation mixture, all enzyme is trapped in the abortive E·KanA·ADP complex, compared to half in the previous experiment.

## 4 Discussion

As pointed out by Gutfreund [23], “rapid reaction techniques are essential for the elucidation of the chemical mechanism of enzyme reactions” and, in particular, “the algebra of steady state kinetics is no substitute for the direct observation of the formation and decomposition of intermediates”. Here, using pre-steady state kinetics methods, we present direct chemical evidence that ADP release is the rate limiting step on the reaction pathway of APH(3′)-IIIa. APH(3′)-Ia and -IIa have also been subject to a pre-steady state study with KanA as the substrate [21,24]. With each enzyme, there was a large transient burst phase of phosphoKanA but the state of the phosphoKanA (free or protein-bound) was not determined.

Further, we show that in the presence of 100 μM KanA, ADP is a powerful inhibitor of APH(3′)-IIIa. In our pre-steady state kinetic study, the decrease of 50% of the ADP burst amplitude and of the steady state rate (Fig. 4A and B) when the same concentration of ADP was added to ATP, suggests that in the presence of KanA, ADP binds to APH(3′)-IIIa with an affinity similar to that of ATP.

Taken together, our results support the proposals of McKay and co-workers [10,22] that the release of ADP occurs from a binary E·ADP complex. However, these proposals were based upon steady state kinetics only, and further, on the use of a coupled enzyme system which perturbs the equilibrium of the APH reaction by removing only one of the products. For this reason, McKay and Wright could not detect the APH(3′)-IIIa inhibition by ADP. The intermediate E·ADP binds tightly KanA and we show that the ADP is released slowly from the ternary E·ADP·KanA complex.

Cornish-Bowden [4] pointed out that a promising strategy in drug design that involves enzymes is to work on uncompetitive rather than the less efficient competitive inhibitors. Here, with APH(3′)-IIIa, the reaction intermediate E·ADP seems to be a good target to develop drugs to inhibit an enzyme that is responsible for the resistance of bacteria to several aminoglycoside antibiotics. Release of ADP is rate limiting, so that a screening assay which tests compounds that reduce the steady state ATPase might be useful for looking for such drugs that bind to E·ADP. As pointed out by Ejim et al. [25], an approach to this end is to combine an antibiotic with a non-antibiotic drug that enhances antibacterial efficacy. To extend the arguments of Cornish-Bowden, we suggest that the non-antibiotic drug should be an uncompetitive inhibitor.
